# Modelling physical contacts to evaluate the individual risk in a dense crowd

**DOI:** 10.1038/s41598-023-31148-z

**Published:** 2023-03-09

**Authors:** Chongyang Wang, Liangchang Shen, Wenguo Weng

**Affiliations:** 1 Department of Engineering Physics, Institute of Public Safety Research, Tsinghua University, Beijing, China; 2 Beijing Key Laboratory of City Integrated Emergency Response Science, Tsinghua University, Beijing, China; 3 China Petrochemical Corporation, Beijing, China

**Keywords:** Psychology and behaviour, Fluid dynamics

## Abstract

Tumble and stampede in a dense crowd may be caused by irrational behaviours of individuals and always troubles the safety management of crowd activities. Risk evaluation based on pedestrian dynamical models can be regarded as an effective method of preventing crowd disasters. Here, a method depending on a combination of collision impulses and pushing forces was used to model the physical contacts between individuals in a dense crowd, by which the acceleration error during physical contacts caused by a traditional dynamical equation can be avoided. The human domino effect in a dense crowd could be successfully reproduced, and the crushing and trampling risk of a microscopic individual in a crowd could be quantitatively evaluated separately. This method provides a more reliable and integral data foundation for evaluating individual risk that shows better portability and repeatability than macroscopic crowd risk evaluation methods and will also be conducive to preventing crowd disasters.

The COVID-19 pandemic starting in 2019 has deeply influencing people’s lifestyle. Mass gathering events were prohibited in the context of urban lockdown^1^. With the increasing rate of vaccination^2^ and the loosening of the epidemic prevention policies^3^, crowd gathering is more and more appearing in life. In crowd gathering situations, because of the backwardness of public facilities and the deficiency of crowd management, some different crowd states, such as turbulence^4–6^ and synchronization^7,8^, may lead to overcrowding or resonant vibrations in crowd activities and may even cause serious casualties^9,10^. Previous research has shown that strong interactions among a dense crowd, especially physical contacts, that are caused by irrational behaviours of individuals occur frequently, which may result in the evolution of the crowd motion state and even crowd disasters^4,11–17^. Current pedestrian dynamical models have been gradually developed into an effective tool to reproduce crowd movement, and also to play an important role in the quantitative risk evaluation of crowd activities^18–20^. Existing dynamical models focus more on the intentional behaviour of pedestrians driven by non-contact interactions between individuals. However, it is necessary to accurately consider unintentional movements resulting from physical contacts between individuals in a high-density crowd^21^. To be more specific, there are three main problems in modelling the physical contacts between individuals in dynamical models. These problems restrict applications from simulating crowd disasters and from developing of a quantitative risk evaluation method.

Firstly, most of the existing dynamical models tend to utilize only the pushing force in modelling physical contacts between individuals^22,23^. However, individuals involved in physical contacts may experience extremely high accelerations exceeding physiological limits due to simplified kinetic equations and idealized numerical calculations. This can be called “acceleration error during individual physical contacts”, objectively requiring excessive contact forces as a guarantee^24^. During collisions, individuals are forced to experience high acceleration in order to avoid overlap and to quickly separate from others, and the contact force and penetration distance will be significantly abnormal if the acceleration is constrained by the physiological reality^25,26^.

Secondly, many investigations suggest that frequent and intense interactions between individuals may have transmitted among the members of a crowd and led to the domino effect in several crowd disasters^27–30^. However, these interactions were mostly based on momentum conservation in existing dynamical models^31^. Therefore, it is difficult to reproduce the domino effect with such models. In fact, a large number of local interactions between individuals who lose balance may be amplified or accumulated among a real crowd due to changes in body posture that are not considered in existing dynamical models^32,33^. Reproducing the domino effect in an accident scenario is difficult with the existing dynamical models^34^, and it is possible to underestimate the crowd risk in simulation.

Thirdly, the mechanism of casualties in crowd disasters has gradually attracted the attention of researchers in this field. These casualties are usually divided into crushing and trampling^35^. Crowd turbulence is usually related to trampling accidents, the risk of which can be quantitatively calculated by parameters such as “crowd pressure”^5,36,37^. However, these risk evaluation methods for a macro crowd are troubled by the selection of the space–time range and the confirmation of the risk thresholds, while risk evaluation methods for a micro individual are related to personal tolerance, which is less affected by external conditions and space–time locations. The existing dynamical models already have the ability to quantitatively calculate the external forces of individuals to evaluate the crushing risk^38^, although they are difficult to use to determine whether individuals lose their balance or fall.

In this work, we model the physical contacts between individuals in a dense crowd based on collision impulses and pushing forces, instead of a simple dynamical equation relying only on the pushing forces.

Our study innovates substantially from other related studies in three aspects. First, this new model can ensure reasonable contact forces and no excessive penetration between individuals with consideration of human physiological acceleration limits. Second, the empirical formula for collision impulses between individuals in different body postures determined from experiments is introduced in this model in order to reproduce the human domino effect in a dense crowd. Third, the method for evaluating the crushing and trampling risk of microscopic individuals in a dense crowd is established depending on the collision impulses and pushing forces obtained in the crowd simulations.

## Results

### Modelling physical contacts

The main source of acceleration error in physical contact in the existing dynamic models is that it completely relies on the imaginary force to change the motion state of an individual in the process of physical contact. Physical contact between individuals is a complex inelastic interactive process. Meanwhile, due to the physiological limits of individual acceleration, contact durations are much longer than those obtained by existing dynamical models, and there is no significant mutual penetration between individuals in reality. Therefore, collision impulses and pushing forces were combined to better model physical contacts in this model. This new model can ensure that the crowd motion process presents the same reasonable kinematic characteristics and laws when the acceleration is constrained by the physiological reality. The contact force generated by an individual during physical contact can also be reasonably estimated. Specifically, physical contacts between individuals were roughly classified into two main categories: collision and pushing. (**I**) When individual *i* had physical contact with individual *j* and they had a tendency to get close to each other in the normal direction, satisfying $({\vec{\mathbf{v}}}_{i} - {\vec{\mathbf{v}}}_{j} ) \cdot ({\vec{\mathbf{p}}}_{i} - {\vec{\mathbf{p}}}_{j} ) < 0$, a passive collision between them would occur; (**II**) when individual *i* had significant contact with individual *j*, satisfying $\left\| {{\vec{\mathbf{p}}}_{i} - {\vec{\mathbf{p}}}_{j} } \right\| < \left( {r_{i} + r_{j} } \right)$, they exerted active pushing towards each other. This new model is more realistic and can provide a more reliable data foundation for the application of risk analysis in crowd activities. Where *v*_*i*_ and *v*_*j*_ represent the velocity before the collision of individual *i* and individual *j* respectively. *p*_*i*_ and *p*_*j*_ represent the positions of individuals *i* and *j*. *r*_*i*_ and *r*_*j*_ represent the radii of individuals *i* and *j*.

#### Collision impulse

The contact force derived from the collision between real individuals cannot be solved analytically by depending on the momentum conservation law in classical physics. However, the instantaneous collision impulse can be used to theoretically update the motion state of an individual during a collision and it is the guarantee of the physiological acceleration limits and the reasonable contact force in a numerical calculation. Generally, the collision impulse Δ*L* can transfer instantly to other individuals to eliminate the relative velocity $$\Delta \overset{\lower0.5em\hbox{$\smash{\scriptscriptstyle\rightharpoonup}$}}{{\mathbf{v}}}_{ij}$$ between individuals and to avoid excessive penetration during the collision. Moreover, the empirical formula of collision impulses was introduced in this model to deal with two different collision forms: (I) Original collision and (II) Secondary collision, representing a collision between individuals who have different body postures ^39^ (*Methods*). The tangential collision can be ignored because an individual often has no time to rotate the body.

#### Pushing force

Traditional pushing forces are only used to remain forces balance of individuals and to avoid further penetration during stable physical contact. Pushing forces between individuals can still be calculated by dynamical equations based on hypothetical forces. However, the values of the two parameters involved in the equations will be much lower than the original values suggested by Helbing et al. ^22^ because pushing forces do not have to provide excessive acceleration in dynamics (*Methods*). In this study, only normal repulsive forces had an obvious compression effect on the human body, while the tangential friction force did not have a significant influence.

#### Dynamical equation

**Figure 1:**
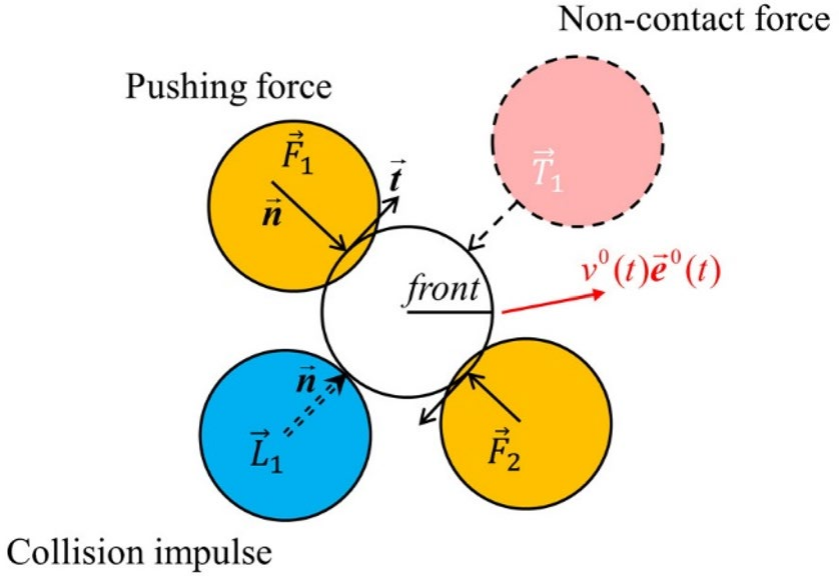
Illustration of interactions of the white pedestrian with others. The physical contacts included a collision impulse (with the blue pedestrian) and pushing forces (with orange pedestrians). Additionally, the dynamics process of the individuals was also subject to the non-contact force (with the pink pedestrian) in the force-based model, while the size $v_{i}^{0} (t)$ and direction $\overrightarrow {{\varvec{e}}}_{i}^{0} (t)$ of the individual desired speed were also determined by the surrounding individual in the velocity-based model.

Considering the physical contacts based on the collision impulses and the pushing forces in this new model, the dynamical equation could be modified as follows:

$$ m_{i} \frac{{d\vec{v}_{i} }}{dt} = m_{i} \frac{{v_{i}^{0} (t)\vec{e}_{i}^{0} (t) - \vec{v}_{i} (t)}}{{\tau_{i} }} + \sum {\vec{T}} + \sum\limits_{j( \ne i)} {\vec{F}_{ij} } + \sum\limits_{j( \ne i)} {\frac{{\Delta \vec{L}_{ij} }}{dt}} + \sum\limits_{W} {\vec{F}_{iW} } + \sum\limits_{W} {\frac{{\vec{L}_{iW} }}{dt}} $$

where *m*_*i*_ represents the mass of the human body *i*.$\tau_{i} = 0.5$ s, representing a certain characteristic time^22^. $\sum {\vec{T}}$ represents the influence of all non-contact forces, which is applicable to the framework of force-based models. The certain desired speed $v_{i}^{0} (t)$ in a certain direction ${\vec{\mathbf{e}}}_{i}^{0} (t)$ can still be calculated by depending on the framework of velocity-based models (Fig. 1). Therefore, this new model can be applied to the framework of two widely used pedestrian dynamical models in order to optimize the physical contacts.

#### Individual risk evaluation

The main purpose of optimizing physical contacts in a pedestrian dynamical model is to acquire more reasonable quantitative interactions and evaluate the individual risk in crowd activities, instead of only simulating the crowd movement.

In crushing accidents, the reason for most casualties and injuries is limited respiration caused by anterior–posterior force and inhibited chest expansion resulting in traumatic asphyxia^40,41^. In a trampling accident, once an individual falls in a dense crowd, a trampling accident will likely be induced and the probability of survival is very low. Therefore, it is reasonable and feasible to consider the individual risk of a trampling accident from the perspective of an individual experiencing unbalanced falling.

#### Crushing risk analysis

The body compression exerted on individuals may result from collision impulses or pushing forces, and the anterior–posterior compression has a significant impact on individuals^42^. Furthermore, the consequence of compression is significantly affected by the average load $f_{i}^{ave}$ exerted on an individual *i* and its duration $D_{i}$ . The empirical formula from the experiments in the literature^43^ can be utilized to calculate the crushing risk:

$$ R_{i}^{crushing} = A\left[ {\ln (f_{i}^{ave} + U)} \right]^{\eta } \times \left[ {\ln (D_{i} + V)} \right]^{\mu } - B \cdot f_{i}^{ave} - W $$

where $A = 5.85 \times 10^{ - 3}$, $B = 0.16 \times 10^{ - 3}$, $U = 1660.03$, $V = 7.28$,$W = 4.94$, $\eta = 3.17$, $\mu = 0.43$, and the range of individual crushing risk satisfies $R_{i}^{crushing} \in \left[ {0,5} \right]$, indicating individual status in a range from comfort to dangerous state. The above equation can be used to update the individual crushing risk during complete simulations (*Methods*).

#### Trampling risk analysis

In a dense crowd, the individual trampling risk can generally be regarded as the probability of falling. However, the tumble probability of an individual is very complex in reality^44^. In general, if an individual is collided with by others from behind in the case of no preparation in advance, it will be very possible for the individual to lose their balance and fall. However, if an individual has collided with another individual from the front side, the possibility of losing their balance and falling is significantly reduced, as the individual can observe and adjust their body posture in advance to actively respond to it. Therefore, the case of a fall to the side and back is not considered in this model. Therefore, the collision impulse coming from the rear and lateral sides of the individual $$\Delta \overset{\lower0.5em\hbox{$\smash{\scriptscriptstyle\rightharpoonup}$}}{{\varvec{L}}}_{r\& l}$$ can be used to evaluate the individual trampling risk:

$$ \Delta \overrightarrow {L} _{{r\& l}}  = \left\{ {\begin{array}{*{20}l}    {\sum {(\Delta \overrightarrow {L} ),} } \hfill & {\overrightarrow {e} .\sum {(\Delta \overrightarrow {L} )\; > \;0} } \hfill  \\    {0,} \hfill & {{others}} \hfill  \\   \end{array} } \right. $$

where $\sum {(\Delta \user2{\mathop{L}\limits^{\rightharpoonup} })}$ represents the total collision impulse exerted on an individual, $\vec{e}$ represents the direction of straight-ahead of the pedestrian.

The combination of collision impulses and pushing forces could faithfully reproduce complex physical contacts between individuals and provide the foundation for evaluating individual risk in a dense crowd. In the following sections, the superiority of this new model is verified by the successive collision simulations compared with the traditional model, and then the risk evaluation method is used to evaluate individual crushing and trampling risks in other typical crowd motion scenarios.

#### Successive collision simulations in a single-person width corridor

**Figure 2:**
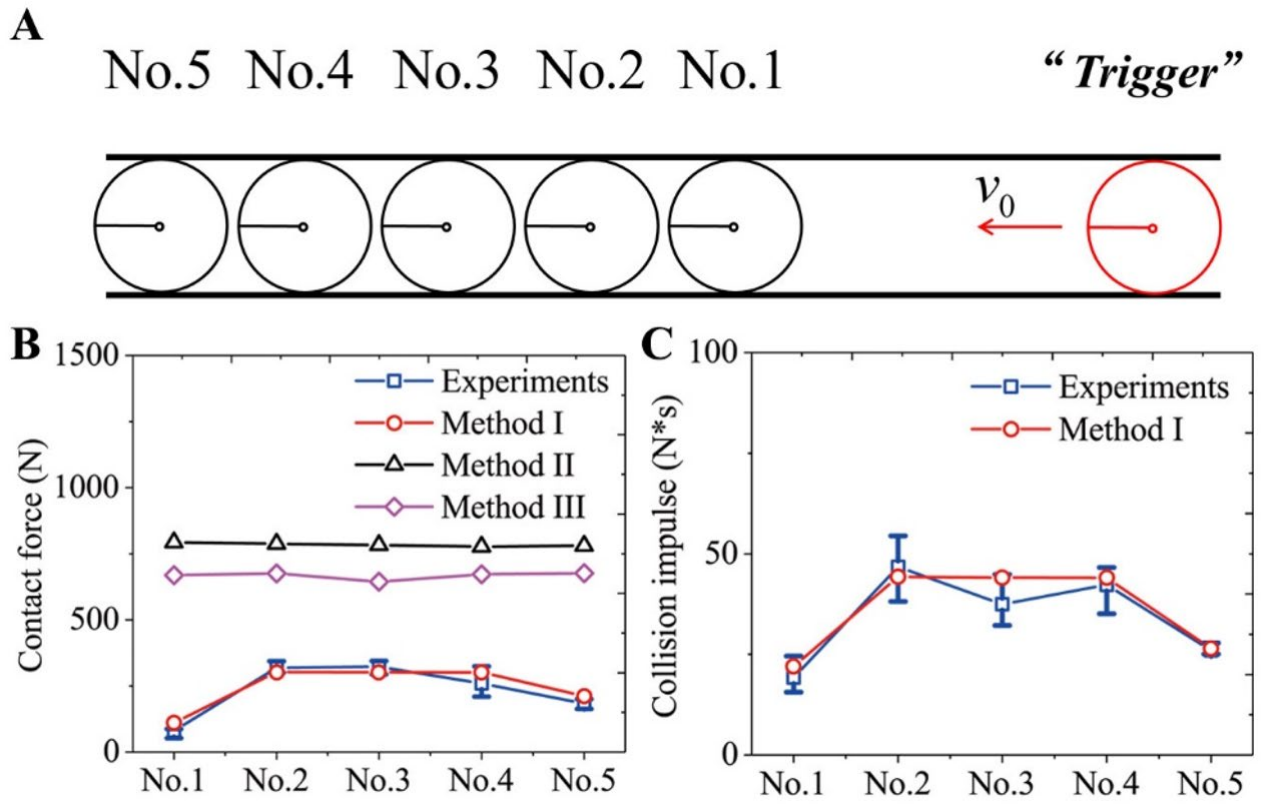
Successive collision simulations in a single-person width corridor. (**A**) Illustration of the simulation scenario. All the individuals were set as circular with r = 0.2 m^31^. The No.1 to No.5 individuals remained stationary with an interval of 0.05 m, and the "Trigger" at the rear moved forward and hit the individual in front with *v*_0_ = 0.35 m/s. In order to be consistent with the experiments, the social forces between individuals and the driving forces of the No. 1 to No. 5 individuals were ignored. Therefore, the movements of the No. 1 to No. 5 individuals were driven only by the physical contacts. (**B**) The contact forces of all individuals were obtained by three methods and experiments. Method I: The physiological acceleration limits of all individuals were assumed to be $a_{upper} = 5\;{\text{m/s}}^{2}$, and the physical contacts between individuals were modeled based on the collision impulses and pushing forces. Method II: The physiological acceleration limits were assumed to be $a_{upper} = 5\;{\text{m/s}}^{2}$, and the physical contacts between individuals were modeled based only on the pushing forces. Method III: No physiological acceleration limits existed, and the physical contacts between individuals were modeled based only on the pushing forces. (**C**) The collision impulses of all individuals were obtained by simulations and experiments. The successive collision impulses transmitted along the queue were not conserved due to the change of individual body posture and they could be amplified or attenuated. In the above simulations, the parameters related to the pushing forces were $k^{\prime} = { 1} \times {10}^{5} {\text{ kg}} \cdot {\text{s}}^{ - 2}$ and $\kappa^{\prime} = 1 \times 10^{5} {\text{ kg}} \cdot {\text{m}}^{ - 1} \cdot {\text{s}}^{ - 1}$. In fact, the simulation results of Method I did not change if the parameters were changed, but the simulation results of Methods II and III changed significantly. If the parameters changed, the contact forces could decrease, but this would lead to excessive penetrations between individuals and aberrant high local densities at the same time. See **SM** for a detailed description.

The experimental data in the same scene was obtained in the literature^39^ as shown in Fig. 2. The contact forces (mainly the collision force in Method I) and the collision impulses obtained by this new model were close to the experimental measurements. If the pushing forces were used alone (Method III), the contact forces obtained in the simulations were significantly higher than the contact forces in the experiments. Moreover, when the physiological acceleration limits of all individuals were considered (Method II), the simulation results were much larger (Fig. 2B). Additionally, the traditional models based on only the pushing forces inevitably depended on the conservation of momentum, so the contact forces were almost the same during the successive collision process. In contrast, the contact forces and the collision impulses calculated by the empirical formula in this new model might fluctuate due to changes in body posture (Fig. 2C).

#### Evacuation simulations in a single-exit room

**Figure 3:**
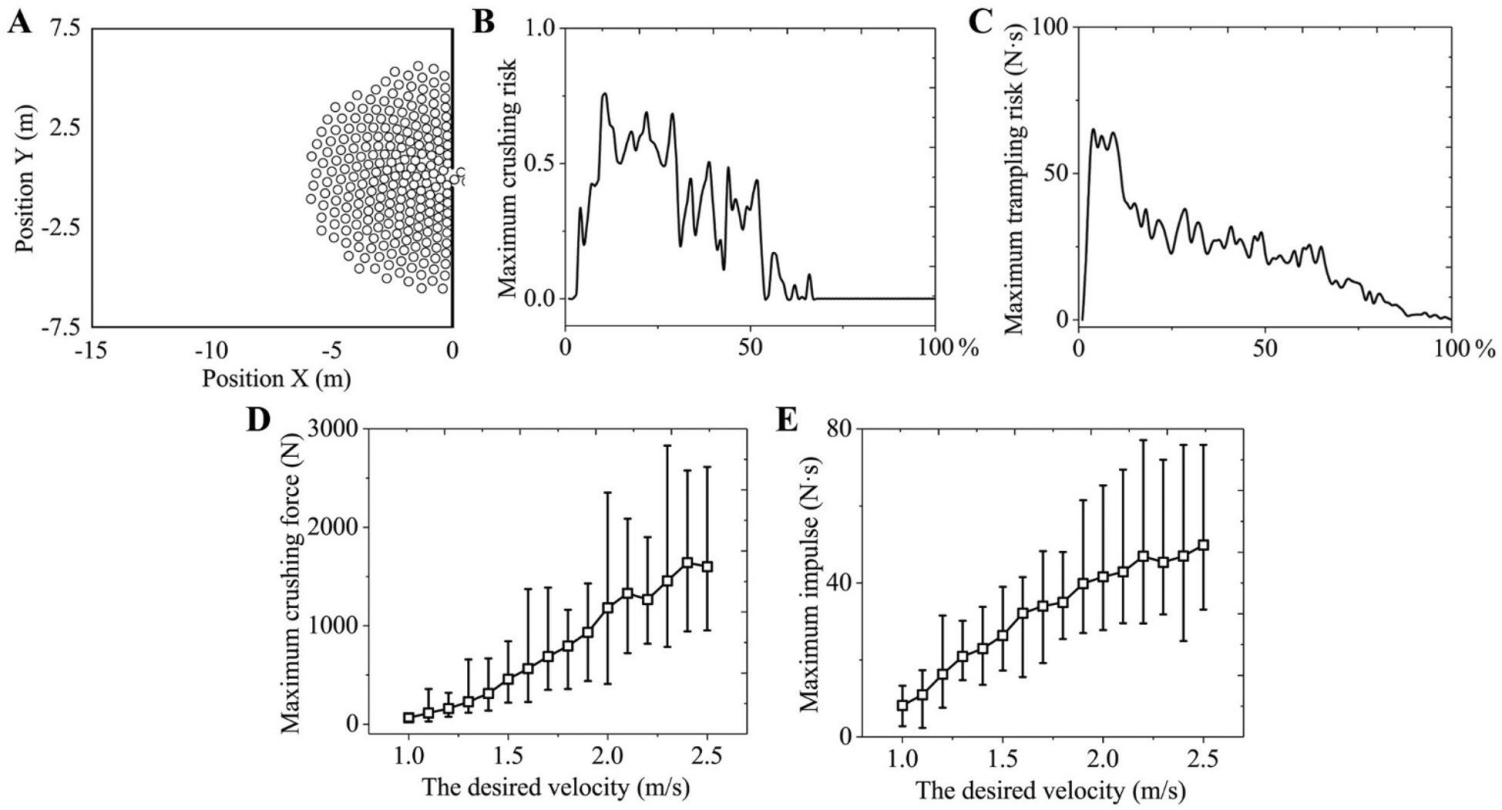
Evacuation simulations in a single-exit room. (**A**) Illustration of the simulation scenario. In these simulations, 300 pedestrians who were distributed uniformly at the initial time would evacuate from a room of 15 × 15 m, and only a 1 m wide exit was located in the middle of one side. The desired velocity of all individuals was 2.5 m/s. All individuals were set as circular with r = 0.2 m. (**B**) The maximum crushing risk of all individuals throughout the entire evacuation process. (**C**) The maximum trampling risk of all individuals throughout the entire evacuation process. (**D**) The maximum crushing force of all individuals during the smooth evacuation phase (5%–30%), which varied with the desired velocity. The error bar indicates the fluctuation range of the maximum crushing force at each desired velocity, and the square point indicates the average value. (**E**) The collision impulse of all individuals during the smooth evacuation phase (5%–30%), which varied with the desired velocity. The error bar indicates the fluctuation range of the maximum collision impulse at each desired velocity, and the square point indicates the average value. In the above evacuation simulations, the physiological acceleration limits were assumed to be $a_{upper} = 5\;{{\text{m}} \mathord{\left/ {\vphantom {{\text{m}} {{\text{s}}^{{2}} }}} \right. \kern-0pt} {{\text{s}}^{{2}} }}$, and the parameters related to the pushing forces were $k^{\prime} = { 2} \times {10}^{{4}} {\text{ kg}} \cdot {\text{s}}^{ - 2}$ and $\kappa^{\prime} = {2} \times 10^{{4}} {\text{ kg}} \cdot {\text{m}}^{ - 1} \cdot {\text{s}}^{ - 1}$.

The simulation results are shown in Fig. 3. When the evacuation reached a smooth phase, the crowd would gather around the exit, forming an arch shape. In addition, physical contact between individuals occurred frequently near the exit, and the physical contacts were relatively more intense when the scale of the congestion was large (Fig. S3A, B). However, in general, the physical contacts between individuals were mostly derived from the lateral direction (Fig. S3C, D). Therefore, the body compression exerted on the human thoracic cavity was not intense and the respiratory function was not significantly inhibited, but it was still possible to cause an individual to lose their balance. When the desired velocity of all individuals was 2.5 m/s (as in a panic situation), the individual crushing risk was still small (Fig. 3B), but the individual trampling risk was enough to cause individuals to fall (Fig. 3C) in special conditions. In addition, the crushing forces and the collision impulses between individuals tended to increase as the desired velocity grew (Fig. 3D, E). That is, when the violent competitive behaviour of individuals in a panic or an excited crowd led directly to an increase in the interaction intensity between individuals, which could more easily cause injuries and deaths.

#### Hajj simulations in the scenario of a 90° turn

**Figure 4:**
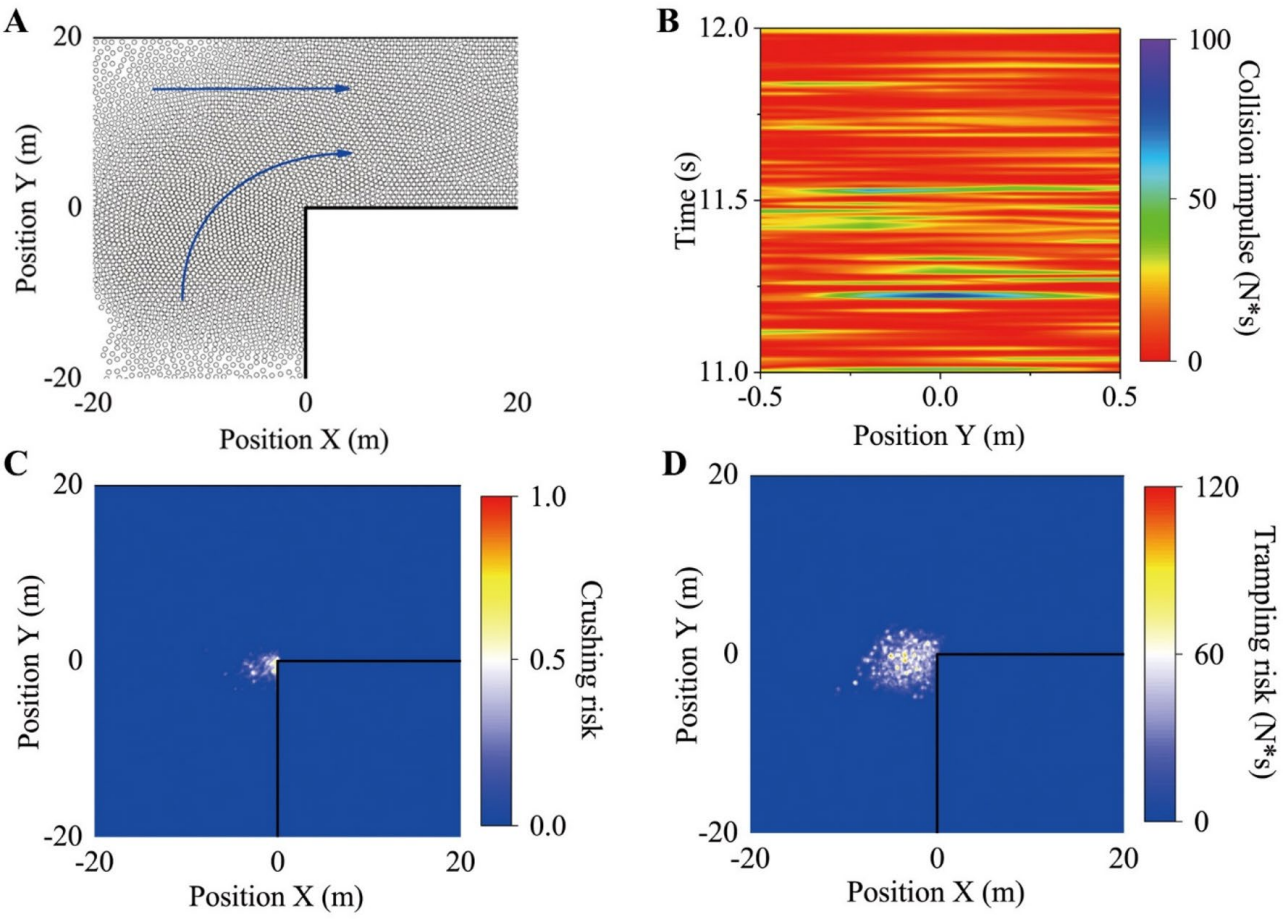
Hajj simulations in the scenario of a 90° turn. (**A**) Illustration of the simulation scenario. In these simulations, 4,746 pedestrians passed through the scene repeatedly, and the pedestrians coming from two main directions would merge together to keep moving, as shown by the solid blue line. Some of these pedestrians had to go through a 90° turn. The desired velocity of all individuals was 1.34 m/s. All individuals were set as circular with r = 0.2 m; (**B**) The space–time diagrams of the collision impulse. The color-coding indicates the propagation phenomenon of the collision impulse generated by the local interaction in the crowd. The smaller slope of the color-coding indicates that the propagation speed of the collision impulse was larger. The change of the color indicates that the collision impulse fluctuated significantly during the propagation process. (**C**) The spatial distribution of the individual crushing risk. (**D**) The spatial distribution of the individual tramping risk. Additionally, the space was divided into grids of ${0}{\text{.2}} \times {0}{\text{.2}}\;{\text{m}}$ in order to describe the spatial distribution characteristics of the individual risks. In order to characterize the individual risks at different locations, the value in each grid was represented by the maximum within 15 s at that location. In the above Hajj simulations, the physiological acceleration limits were assumed to be $a_{upper} = 5{{\;{\text{m}}} \mathord{\left/ {\vphantom {{\;{\text{m}}} {{\text{s}}^{{2}} }}} \right. \kern-0pt} {{\text{s}}^{{2}} }}$, and the parameters related to the pushing forces were $k^{\prime} = { 2} \times {10}^{{4}} {\text{ kg}} \cdot {\text{s}}^{ - 2}$ and $\kappa^{\prime} = {2} \times 10^{{4}} {\text{ kg}} \cdot {\text{m}}^{ - 1} \cdot {\text{s}}^{ - 1}$.

The results are shown in Fig. 4. During the simulations, the laminar, stop-and-go, and turbulence flows could be observed in the whole space (Fig. S6A). However, the physical contacts were only possible in the local area near the turn, where the individual crushing risk and the trampling risk were relatively high (Fig. 4C, D). To be more specific, the obvious anterior–posterior compression would be exerted on the thoracic cavity of individuals (Fig. 7A), and the instantaneous compression strength could exceed the tolerance of individuals (Fig. S5C). However, the scale of congestion was small, that is, contact duration was not long; thus, the individual crushing risk was still low. In addition, because of the turning and merging, the collision impulses were mostly toward the right side of the body (Fig. S7B), and the trampling risk was sufficient to cause some individuals to fall (Fig. S5D). Moreover, once a local collision impulse was generated in the dense crowd, it was very likely to transmit among the crowd. In special cases, the impulse would be amplified constantly during the propagation, resulting in the human domino effect (Fig. 4B). Finally, a strong collision impulse could lead to an individual losing balance and falling, and then causing a crowd disaster.

#### Love Parade simulations in a corridor

**Figure 5:**
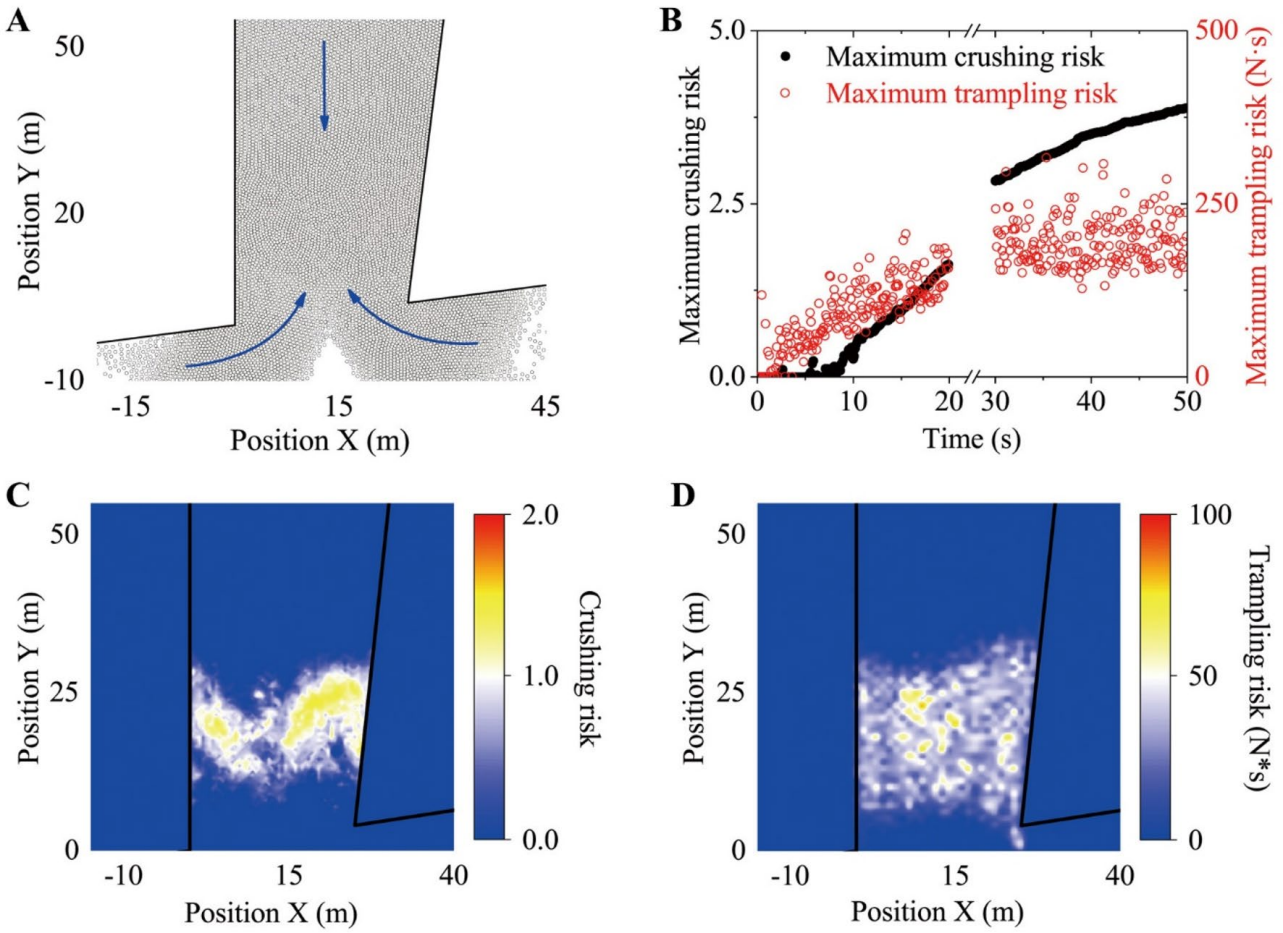
Love Parade simulations in a corridor. (**A**) Illustration of the simulation scenario. In these simulations, 12,200 pedestrians were divided into three sections and they entered the scene separately from the three entrances of the corridor. The pedestrians entering the corridor from the two entrances below merged together to move upwards, while the pedestrians entering the area from the entrance above kept moving downwards, as shown by the solid blue line. Finally, two opposite streams of pedestrians jammed into the corridor. The desired velocity of all individuals was 1.34 m/s. All individuals were set as circular with r = 0.2 m. (**B**) The maximum crushing and trampling risk of all individuals during 50 s (0–20 s, 30–50 s) after the upward and downward streams of the pedestrians merged together. (**C**) The spatial distribution of the individual crushing risk. (**D**) The spatial distribution of the individual tramping risk. Additionally, the space was divided into grids of ${0}{\text{.5}} \times {0}{\text{.5}}\;{\text{m}}$ in order to describe the spatial distribution characteristics of individual risks. In order to characterize the individual risks at different locations, the value in each grid was represented by the maximum for 10 s–20 s after the two streams of pedestrians merged together at that location. In the above Love Parade simulations, the physiological acceleration limits were assumed to be $a_{upper} = 5\;{{\text{m}} \mathord{\left/ {\vphantom {{\text{m}} {{\text{s}}^{{2}} }}} \right. \kern-0pt} {{\text{s}}^{{2}} }}$, and the parameters related to the pushing forces were $k^{\prime} = { 2} \times {10}^{{4}} {\text{ kg}} \cdot {\text{s}}^{ - 2}$ and $\kappa^{\prime} = {2} \times 10^{{4}} {\text{ kg}} \cdot {\text{m}}^{ - 1} \cdot {\text{s}}^{ - 1}$.

The results are shown in Fig. 5. In the congestion area, the crowd was in turbulent status (Fig. S9C, S10), and physical contact between individuals including collision and pushing occurred frequently. These local interactions could continue to accumulate and transmit, so the individual crushing and trampling risks were relatively high (Fig. 5C, D). To be more specific, the obvious anterior–posterior compression was also exerted on the individuals (Fig. S11A), and the instantaneous compression strength was very high (Fig. 8C). Additionally, it was difficult for the crowd to effectively evacuate in a short time once the crowd was jammed in the corridor. Therefore, the individuals had to be subjected to intense compression continuously, and the individual risk continued to rise rapidly. As shown in Fig. 5B, some individuals could be unable to endure this overcrowding situation just after the occurrence of the crowd congestion lasting 50 s. In addition, individuals in the congestion area could also be subjected to very strong longitudinal collision impulses (Fig. S11B). In the case of high local density (Fig. S8A), the collision impulses could easily induce the human domino effect and they could become enlarged through propagation. The extent of trampling risk might eventually lead to an individual losing balance and falling (Fig. 5B).

## Discussions

Physical contact between individuals in a dense crowd could be well simulated based on the model proposed in this paper. This new model could ensure reasonable contact forces and no excessive penetration between individuals, with consideration of the human physiological acceleration limits (Fig. 2). Additionally, it could be applied to the framework of force-based models and velocity-based models and effectively optimize the physical contacts theoretically and quantitatively. The collision impulses were introduced into the traditional model framework to deal with the rapid penetration between individuals in order to avoid the “acceleration error”, and to provide an effective way to reproduce the domino effect that might occur in real crowd disasters.

Firstly, the “acceleration error” of the traditional method that was based only on pushing forces restricted the development of pedestrian dynamical models in risk assessment. The main goal of the new model is to quantitatively and reasonably calculate the interactions involving physical contacts, which are the necessary foundation for the development from evaluating crowd risk with a macroscopic crowd movement state to evaluating individual risk with the external forces and impulses. From the micro-individual perspective, the anterior–posterior crushing forces can be used to evaluate the individual crushing risk, while the rear and lateral collision impulses can be used to evaluate the individual trampling risk (Fig. 3). The dangerous areas obtained by this new model were consistent with the empirical scenes (Figs. 4 and 5). Since the individual risk threshold was only related to personal tolerance and balance ability, it was not necessary to consider the external environment in the calculation. The important advantage of this new model is mainly reflected in the application to the risk assessment of crowd activities.

Secondly, the framework of traditional models based only on pushing forces depended entirely on the momentum conservation law, and it considered no influence from the changes in human body posture during physical contact in a dense crowd. However, the physical contact between individuals would be changed significantly when individuals lost their balance, and the two-dimensional momentum conservation law would not be satisfied. After the corresponding empirical formula of the collision impulses was introduced, this model could be used to effectively describe physical contacts between individuals in unstable postures, in order to conveniently reproduce the domino effect that could not be achieved by the momentum conservation law (Fig. 4B). The collision impulses produced by local interactions in a dense crowd was likely to trigger the domino effect, which is one of the most common dangerous phenomena in real crowd disasters. The optimization of the physical contacts in the new model would provide important support for simulating real crowd movements, especially for reproducing crowd disasters.

In the future, the systematic experimental data including the crushing forces and collision impulses in a more complex crowd evacuation scenario are necessary to further verify the simulation results of this new model. In addition, biomechanical experiments should be carried out to obtain a large amount of basic data about human tolerance and balance ability, which is useful for improving the cognitive level of individual risk and conducting an individual risk assessment. This will be an important prerequisite for evaluating micro-individual risk in crowd activities.

## Methods

### Collision impulse

#### Original collision

If two individuals in an upright posture collided with each other, it could be called original collision. When individual *i* collided with individual *j* in a dense crowd due to the inability to avoid each other, the original collision impulse was solved as follows:

$$ \Delta \overrightarrow {{\varvec{L}}}_{ij} \; = \;\frac{{m_{i} m_{j} }}{{m_{i} \; + \;m_{j} }}\left( {1 + e} \right)\Delta v_{ij}^{n} \cdot \overrightarrow {{\varvec{n}}}_{ij} $$

where *m*_*i*_ and *m*_*j*_, respectively, represent the mass of individual *i* and individual *j*; the relative velocity in the normal direction is expressed as $\Delta v_{ij}^{n} = ({\vec{\mathbf{v}}}_{j} - {\vec{\mathbf{v}}}_{i} ) \cdot {\vec{\mathbf{n}}}_{ij}$, and ${\vec{\mathbf{n}}}_{ij} = {{\left( {{\vec{\mathbf{p}}}_{i} - {\vec{\mathbf{p}}}_{j} } \right)} \mathord{\left/ {\vphantom {{\left( {{\vec{\mathbf{p}}}_{i} - {\vec{\mathbf{p}}}_{j} } \right)} {\left\| {{\vec{\mathbf{p}}}_{i} - {\vec{\mathbf{p}}}_{j} } \right\|}}} \right. \kern-0pt} {\left\| {{\vec{\mathbf{p}}}_{i} - {\vec{\mathbf{p}}}_{j} } \right\|}}$ is expressed the unit direction vector of individual *i* to individual *j*, and *v*_*i*_ and *v*_*j*_, represent the velocity before collision of individual *i* and individual *j* respectively. The elastic restitution coefficient was assumed to be *e* = 0.8.

#### Secondary collision

If a collision impulse $$\Delta \overset{\lower0.5em\hbox{$\smash{\scriptscriptstyle\rightharpoonup}$}}{{\varvec{L}}}_{0}$$ made individual *i* lose their balance and there was an individual *k* around individual *i*, then the secondary collision could occur for the case where individuals collided with each other in an unstable posture. Depending on the empirical formula obtained in the experiments ^39^, the secondary collision impulse could be approximately solved as:

$$ \Delta \vec{L}_{jk} = \left[ {a\exp^{{{{pm_{j} d_{jk} } \mathord{\left/ {\vphantom {{pm_{j} d_{jk} } {(\Delta \vec{L}_{0} \cdot \vec{n}_{jk} )}}} \right. \kern-0pt} {(\Delta \vec{L}_{0} \cdot \vec{n}_{jk} )}}}} + b\exp^{{{{qm_{j} d_{jk} } \mathord{\left/ {\vphantom {{qm_{j} d_{jk} } {(\Delta \vec{L}_{0} \cdot \vec{n}_{jk} )}}} \right. \kern-0pt} {(\Delta \vec{L}_{0} \cdot \vec{n}_{jk} )}}}} + \delta } \right] \cdot \left( {\Delta \vec{L}_{0} \cdot \vec{n}_{jk} } \right) \cdot \vec{n}_{jk} $$

where *m*_*j*_ represents the mass of the human body* j*, *d*_*jk*_ represents the distance between the mass center of individuals* j* and *k*, $${\vec{\mathbf{n}}}_{jk}$$ represents the normal direction vector from individual *j* to *k*, and the constant $$a = 0.32$$, $$b = 1.33$$, $$p = 1.21$$, $$q = - 4.99$$. According to the experiments^43^, there were slight differences in$${\raise0.7ex\hbox{${\Delta {\varvec{L}}_{jk} }$} \!\mathord{\left/ {\vphantom {{\Delta {\varvec{L}}_{jk} } {\Delta {\varvec{L}}_{0} }}}\right.\kern-0pt} \!\lower0.7ex\hbox{${\Delta {\varvec{L}}_{0} }$}}$$ at different positions in the queue. Therefore, in this paper, $$\delta$$ was set to obey the normal distribution N(− 0.2, 0.05) if there were no pedestrians restricting the movement of the collided individual within the available range, and $$\delta$$ obeyed the normal distribution N(− 0.2, 0.05) in other cases.

### Pushing force

$$ \overrightarrow {{\varvec{F}}}_{ij} \; = \;k^{\prime}g(r_{ij} - d_{ij} )\overrightarrow {{\varvec{n}}}_{ij} + \kappa^{\prime}g(r_{ij} - d_{ij} )\Delta v_{ji}^{t} \overrightarrow {{\varvec{t}}}_{ij} $$

where $r_{ij} = r_{i} + r_{j}$ represents the sum of two individual radii. If there is no physical contact between individuals, $g(x) = 0$, otherwise $g(x)\; = \;x$. $\Delta v_{ji}^{t} = ({\vec{\mathbf{v}}}_{j} - {\vec{\mathbf{v}}}_{i} ) \cdot {\vec{\mathbf{t}}}_{ij}$ expresses the tangential velocity difference. $k^{\prime}$ and $\kappa^{\prime}$ represent the correction coefficients of the normal repulsive force and the tangential friction. In addition, the pushing forces between the individuals and the wall satisfy:

$$ \overrightarrow {{\varvec{F}}}_{iW} \; = \;k^{\prime}g(r_{i} - d_{iW} )\overrightarrow {{\varvec{n}}}_{iW} + \kappa^{\prime}g(r_{i} - d_{iW} )\left( {\overrightarrow {v}_{i} \cdot \overrightarrow {{\varvec{t}}}_{iW} } \right)\overrightarrow {{\varvec{t}}}_{iW} $$

where $d_{iW}$ represents the distance between the individual mass center and the wall, and ${\vec{\mathbf{t}}}_{iW}$ represents the tangential direction of the wall surface.

#### Definition of the force variables

The contact force is defined as the vector sum of the collision forces and pushing forces exerted on an individual and it drives the unintentional movement of an individual during physical contact.

The crushing force is also a vector. Its scale indicates the strength of the body compression caused by collision forces (See Collision force in the Supplementary material for the calculation method) and normal pushing forces (See Pushing force in the Methods section for the calculation method) and its direction indicates the predominant direction of the body compression.

In general, the crushing force is not equal to the contact force, which means that even if an individual reaches force equilibrium, body compression will also exist. That is, when the contact force is zero, the crushing force will not necessarily be zero. In addition, the anterior–posterior component of the crushing force exerted on an individual will contribute to the individual crushing risk.

## Supplementary Information

Supplementary Information.

## Data Availability

The datasets used and/or analyzed during the current study available from the corresponding author on reasonable request.
