# Supplementary material for: Modelling physical contacts to evaluate the individual risk in a dense crowd
Source: Sci Rep. 2023 Mar 9;13:3929. doi: 10.1038/s41598-023-31148-z (PMC9995744; doi:10.1038/s41598-023-31148-z)
Supplement: Supplementary file 1 — Supplementary Information. [file 41598_2023_31148_MOESM1_ESM.docx]

Supplementary material for

Modelling physical contacts to evaluate the individual risk in a dense crowd

Chongyang Wang, Liangchang Shen, Wenguo Weng

Wenguo Weng

Email: wgweng@tsinghua.edu.cn

**This PDF file includes:**

Supplementary text

Figs. S1 to S11

References for SI reference citations

Supplementary Information: Modelling physical contacts

**Collision impulse.** There may be two different forms of the collision between individuals in a dense crowd.

**(I) Original collision.** If a collision occurs between an individual *i* and a wall *W*, the collision impulse can be expressed by $\Delta\vec{\mathbf{L}}_{iW}=m_{i}\Delta v_{iW}^{n}\cdot\vec{\mathbf{n}}_{iW}$, where $\Delta v_{iW}^{n}=(-\vec{\mathbf{v}}_{i})\cdot\vec{\mathbf{n}}_{iW}$, and $\vec{\mathbf{n}}_{iW}$ represents the unit direction vector from a wall *W* to an individual *i* that is perpendicular to the wall space. If an individual is not yet aware of imminent collision from the rear and lateral direction, the individual will probably tilt to the side front forward because of losing balance by collision impulse. Overall, if individuals are subjected to collision impulses from the front side, the possibility of an unstable tip occurring is significantly lower because they can observe beforehand and adjust their body posture to respond actively. Therefore, the situation of a tip side backward is not considered by this model.

**(II) Secondary collision.** Because the distance between individuals in a dense crowd is very small, this form of collision has a very significant feature, which is that physical contact does not occur for an individual in an unstable inclined posture relative to others.

After a secondary collision, an individual *j* will be subjected to a physiological balance posture. If there are no other pedestrians around individual *j*, the balanced posture of individual *j* can be recovered by a step movement, and secondary collision will not occur.

In fact, a secondary collision occurs in an unstable tip attitude that does not comply with the traditional conservation of momentum (1, 2), which is also the reason why a human domino effect may occur in a dense crowd. The surge phenomenon in a real crowd is not just a simple impulse transmission process. The key to crowd accidents lies in the dynamic change of impulse in the transmission process, especially in the accumulation effect of the human domino effect. Therefore, if the original collision in an upright posture is considered exclusively, the collision process in a real dense crowd cannot be presented accurately.

**Collision force.** In a collision between individuals, although the collision impulse can be used to achieve the update of the individual motion state, it is still necessary to calculate the collision force during the impulse transmission in order to measure the extent of body compression produced by a collision between individuals. However, since the collision force between real human bodies cannot be directly solved from the theory, accurate quantitative modelling must have be based on relevant empirical data, and the collision force can be indirectly solved by the collision impulse between individuals.

In the physiological successive collision experiment, the numerical relationship satisfied by the secondary collision force pulse was measured, and the maximum value of the secondary collision force pulse could be solved as follows:

$f_{Max}=\left[\Gamma+\beta\left(\Delta L\times d_{jk}^{2}\right)\right]^{2}$,

where constant $\Gamma=10.36814$, $\beta=0.78479$. Additionally, after data statistics, it can be found that the duration of a secondary collision force pulse is ~0.5 s. This is likely to be similar to the original collision force pulse duration, so the original collision force pulse can be simply assumed to satisfy the following equation:

$f_{Ave}=\Delta L/\alpha=f_{Max}/2$,

where $f_{Ave}$ and $f_{Max}$ represent the average and maximum values of the original collision force pulse, respectively, and *α* represents the duration of the original collision force pulse, assumed to be $\alpha\text{ }=0.5\pm0.1$ s.

**Supplementary Information: Individual risk evaluation method**

In a trampling accident, it is generally considered that the individual instability and fall is the root cause of the trampling accident, but in a crushing accident, external pressure usually acts on the chest of a human body, causing the individual to suffer from breathing and suffocation.

**Crushing risk analysis.** For a single individual *i*, if physical contact exists with another single individual *j*, the individual may be subjected to an instantaneous collision impulse $\Delta\vec{\mathbf{L}}_{ij}$ or an axial direction pushing force $\vec{\mathbf{F}}_{ij}^{n}$. The contribution of the crushing risk for an individual *i* from body compression between individuals can be expressed as:

$\Delta L_{ij}^{r}=\Delta\vec{\mathbf{L}}_{ij}\cdot\vec{\mathbf{e}}_{i}$,

$F_{ij}^{r}=\vec{\mathbf{F}}_{ij}^{n}\cdot\vec{\mathbf{e}}_{i}$,

where the instantaneous expected velocity direction $\vec{\mathbf{e}}_{i}$ is assumed to be the direct front of the body of an individual. The contribution of all body compression applied by all individuals who have physical contact with the individual *i* to the crushing risk of individual *i* can be expressed as:

$Q_{i}^{r}=\frac{1}{2\Delta t}\left[\sum_{j\in\Omega} \left(\left|F_{ij}^{r}\Delta t\right|+\left|\Delta L_{ij}^{r}\right|\right)+\left|\sum_{j\in\Omega} \left(F_{ij}^{r}\Delta t+\Delta L_{ij}^{r}\right)\right|\right]$,

where Δ*t* represents the time step in simulation, and the collection Ω consists of all individuals who have physical contact with the individual i and who apply body compression. $Q_{i}^{r}$ can be used to update the average load. Its duration suffers from the process of successive physical contact with the individual *i*, and the crushing risk from individual *i* is evaluated at the end. If $Q_{i}^{r}=0$, it means that physical contact does not occur between individual *i* and any other individuals. In the modelling process, the external load and duration of an individual will change over time, if the average load $f_{i}^{ave}(t)$ and its duration $D_{i}(t)$ subjected to the individual at any time *t* is known. Then at *t* + ∆*t*, the average load $f_{i}^{ave}(t+\Delta t)=\left[f_{i}^{ave}(t)\cdot D_{i}(t)+Q_{i}^{r}(t+\Delta t)\cdot\Delta t\right]/\left(D_{i}(t)+\Delta t\right)$ and its duration $D_{i}(t+\Delta t)=D_{i}(t)+\Delta t$ are borne by the individual.

**Supplementary Information: Results and Discussions**

**Successive collision simulations in a single-person width corridor.** In the simulation of a successive collision process in a single-person width corridor, it can be found that when the parameters $k'=\text{ 1}\times10^{5}\text{kg}\cdot\text{s}^{-2}$ and $\kappa'=1\times10^{5}\text{kg}\cdot\text{m}^{-1}\cdot\text{s}^{-1}$ are used to express the pushing force, the contact force calculated by the method is significantly lower than that based only on pushing forces. However, the parameters $k'$ and $\kappa'$ in the pushing force expression have been frequently changed by some researchers (3). The interaction intensity of individual contact in crowd movement can be affected significantly, and thus the motion state of a dense crowd is affected. We used two cases for a detailed discussion.

(1) For the case when the "Trigger" at the rear moved forward at a speed of *v_0_* = 0.35 m/s and struck the front individual, when the parameters $k'$ and $\kappa'$ were lowered, the modelling results based only on the pushing forces (**Method II and III**) are shown in Fig. S1. Although the contact force and the acceleration experienced by an individual decreased with the decrease of the parameters $k'$ and $\kappa'$ (Fig. S1A, S1C), the penetration distance during the collision between individuals increased (Fig. S1B). Additionally, since the initial speed of the "Trigger" was low and the increase of the penetration distance was still very limited, the impact on the phenomenon of macroscopic crowd movement may not have been significant. (2) For the case when the "Trigger" at the rear moved forward at a speed of *v_0_* = 1.4 m/s and struck the front individual, the simulation results obtained by simply relying on the pushing force method (**Method II and III**) are shown in Fig. S2. Similarly, if no physiological acceleration limits existed (**Method III**), the contact force an individual was subjected to and the acceleration experienced by an individual would decrease significantly with the decrease of the parameters $k'$ and $\kappa'$ (Fig. S2A, S2C), but the infiltration distance increased significantly when a collision between individuals occurred (Fig. S2B). For example, when $k'\text{=}5\times10^{3}\text{ kg}\cdot\text{s}^{-2}$, the contact force an individual was subjected to was closer to the simulation result obtained using this method (**Method I**), but the acceleration experienced by an individual was still approximately as high as 10 m/ s^2^. The infiltration distance between individuals could be greater than 0.12 m (the radius of an individual is only 0.2 m), which would lead to an abnormal increase in local crowd density during large-scale crowd simulation, which in turn led to simulation results that were contrary to reality. Meanwhile, if the physiological acceleration limits of individuals (**Method II**) were considered, the contact force and penetration distance would further increase. There was a limitation when the interaction of individuals in physical contact was quantitatively described based only on the pushing forces. Overall, the reasonable contact force and the penetration distance could not be accounted for simultaneously, and if the physiological acceleration limits of real human bodies were considered, more trouble was caused for quantitative characterization.

**Evacuation simulations in a single-exit room.** In the simulation of the crowd evacuation process in a single exit room, and in the scenario where the individual expected speed was 1.5 m/s, this method (**Method I**) and the method of relying solely on a pushing force (**Method II and III**) were used for simulation. The result is shown in Fig. S3. If the physiological acceleration limits were not considered, and the physical contacts between individuals were simulated based only on the pushing forces (**Method III**), the result of the crushing force at most times was only slightly larger than the simulation result of this method (**Method I**). However, once the acceleration limits of actual human bodies were considered, the simulation results based only on pushing forces increased significantly (**Method II**), as shown in Fig. S3A. The simulation results obtained by this method were close to those of measurement results (4) and close to the simulation results in the crowd motion scenes of other scholars. During the entire evacuation process, as the congestion scale gradually eased, the collision impulse gradually decreased (Fig. S3B). More importantly, during the crowd evacuation process, physical contact between most individuals only occurred in the side direction, that is, the crushing force and the collision impulse of an individual were concentrated in the side direction of the body (Fig. S3C, S3D). Therefore, the squeezing effect of the chest cavity of the human body was not significant, but the individual might have been in an unstable condition.

The macroscopic spatial distribution of the crowd evacuation process is shown in Fig. S4. The crowd local density near the bottleneck was the largest, more than 6 people/m^2^. The individuals were arranged very closely and the phenomenon of a typical arch distribution was exhibited (Fig. S4A). Simultaneously, only individuals close to the bottleneck could move quickly, and physical contact might occur between the individuals who moved quickly and other individuals. At other locations, the movement speed of an individual was very small (Fig. S4B), so only the crushing force and the collision impulse that individuals were subjected to close to the bottleneck were most significant (Fig. S4C, S4D), and the location was consistent with the region with the highest local density. Similarly, the crushing risk and trampling risk were relatively high when individuals were close to the bottleneck (Fig. S4E, S4F). Because the individuals were in a normal evacuation process simultaneously, the competitive behaviour was not strong, the individual risk level was low, and the individual almost did not feel any discomfort.

**Hajj simulations in the scenario of a 90° turn.** The method proposed in this paper was used to simulate the crowd motion scene of the Hajj pilgrimage. The macroscopic spatial distribution of the crowd motion state is shown in Fig. S5. It can be found that the local density of the crowd at the turn was relatively high, close to 7 people/m^2^, which indicated that the individuals at the location in the simulation were already close to each other (Fig. S5A). Simultaneously, with the crowd motion process toward the corner, the speed was gradually reduced, the individual speed was obviously lower than that of other positions when approaching the turning point, and the local congestion was formed by pedestrians at this location (Fig. S5B). In this process, three typical crowd movements, namely the laminar flow state, stop-and-go state, and turbulence state, could be observed, as shown in Fig. S6A. In particular, in the local congestion area, the turbulent motion of the crowd could be reproduced, as shown in Fig. S6B and Fig. S6C, which was a typical feature of the turbulent state.

Meanwhile, physical contact would inevitably occur between an individual and others, and the individual would be subjected to a significant crushing force and a collision impulse (Fig. S5C, S5D). The local congested area was also the turbulent area, which indicated that the collision and pushing would occur frequently for an individual in a turbulent state with the surrounding individuals, and the collision impulse and pushing force between them would continue to be generated and might be transmitted in the crowd.

**Love Parade simulations in a corridor.** The method proposed in this paper was used to simulate the crowd motion scene at the Love Parade 2010. The macroscopic spatial distribution of the crowd motion state is shown in Fig. S8. It can be found that when the crowd was hedged in the corridor, congestion formed, and the crowd local density at the location of the congestion was close to 10 people/m^2^, which was close to the limit of the local density. The individuals were already close to each other and physical contact would inevitably occur (Fig. S8A). The local speed at the location of the congestion was significantly smaller than the speeds at other locations within the space, and most of the individuals in the congested position were almost at a standstill (Fig. S8B). The crowd movement at the congestion location was very chaotic. A significant turbulent movement state is shown (Fig. S9B) and a typical turbulence feature is shown in Fig. S10. There was still laminar flow motion when the crowd did not reach the congested position (Fig. S9A). At the same time, hedging people generated large-scale persistent congestion, physical contact would inevitably occur between an individual and others, and crushing forces and collision impulses were received significantly by individuals (Fig. S8C, S8D). Simultaneously, the congested area was the same as the turbulent area, which also indicated that collisions and pushing would occur frequently for individuals in a turbulent state with the surrounding individuals.


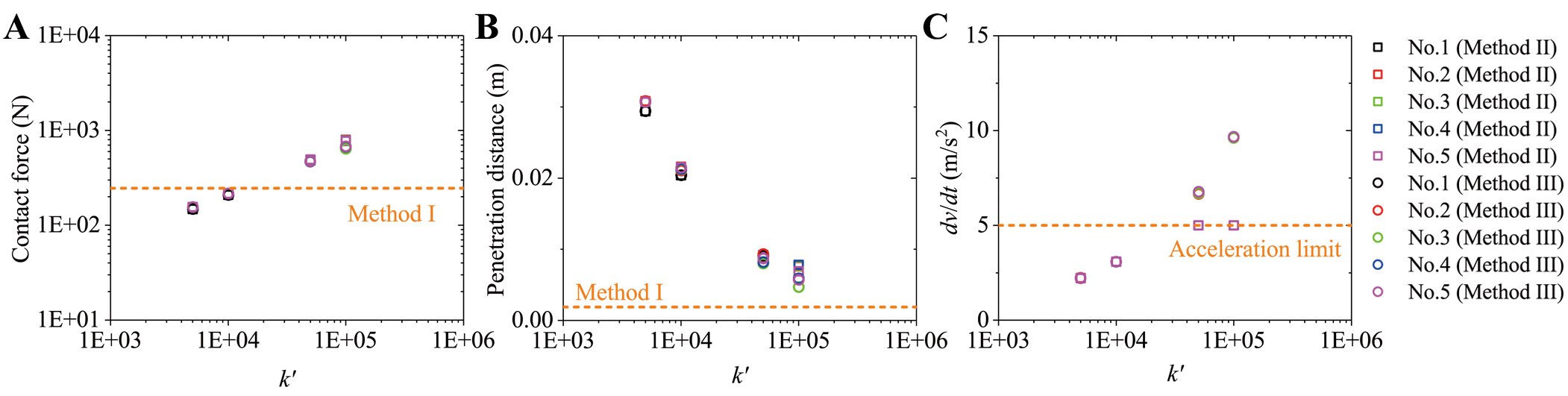


Fig. S1. Successive collision simulations in a single-person width corridor under different parameters $k'$. The initial speed of the “Trigger” was *v*_0_ = 0.35 m/s, and the value of $\kappa'$ was equal to$k'$. Method I: The physiological acceleration limits of all individuals were assumed to be $a_{upper}=5 \text{m/s}^{2}$, and the physical contacts between individuals were modeled based on the collision impulses and pushing forces. Method II: The physiological acceleration limits of all individuals were assumed to be $a_{upper}=5 \text{m/s}^{2}$, and the physical contacts between individuals were modeled based only on the pushing forces. Method III: No physiological acceleration limits existed, and the physical contacts between individuals were modeled based only on the pushing forces. (A) The contact forces included collision forces and pushing forces an individual was subjected to. (B) The infiltration distance of the individual during physical contact. (C) The acceleration the individual was subjected to during physical contact.


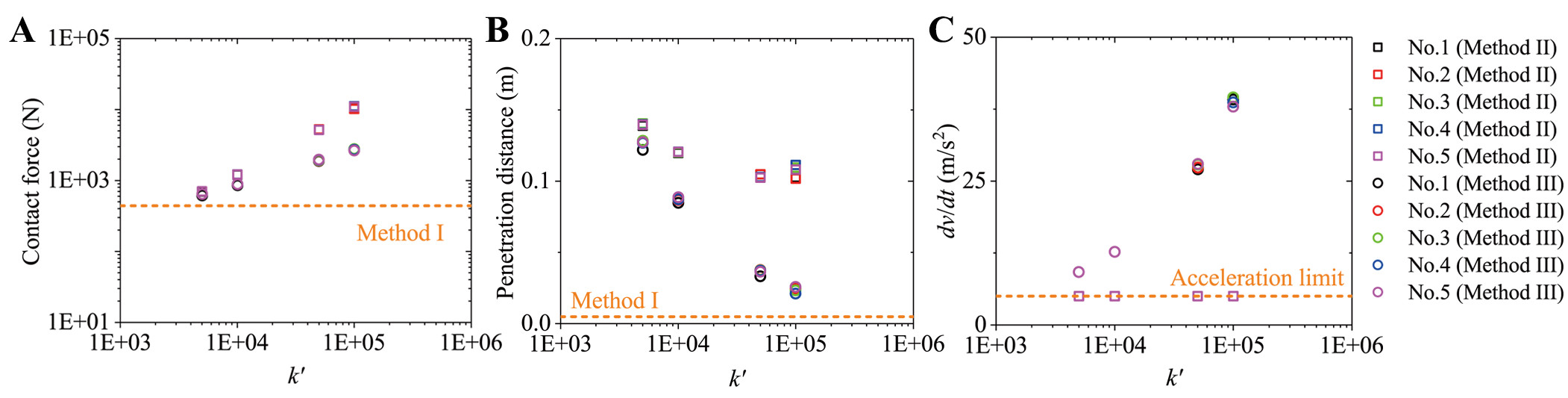


Fig. S2. Successive collision simulations in a single-person width corridor under different $k'$ Parameters. The initial speed of the “Trigger” was *v*_0_ = 1.4 m/s, and the value of $\kappa'$ was equal to$k'$. Method I: The physiological acceleration limits of all individuals were assumed to be $a_{upper}=5 \text{m/s}^{2}$, and the physical contacts between individuals were modeled based on the collision impulses and pushing forces. Method II: The physiological acceleration limits of all individuals were assumed to be $a_{upper}=5 \text{m/s}^{2}$, and the physical contacts between individuals were modeled based only on the pushing forces. Method III: No physiological acceleration limits existed, and the physical contacts between individuals were modeled based only on the pushing forces. (A) The contact force the individual was subjected to, including the collision force and the pushing force. (B) The infiltration distance the individual was subjected to during physical contact. (C) The acceleration the individual was subjected to during physical contact.


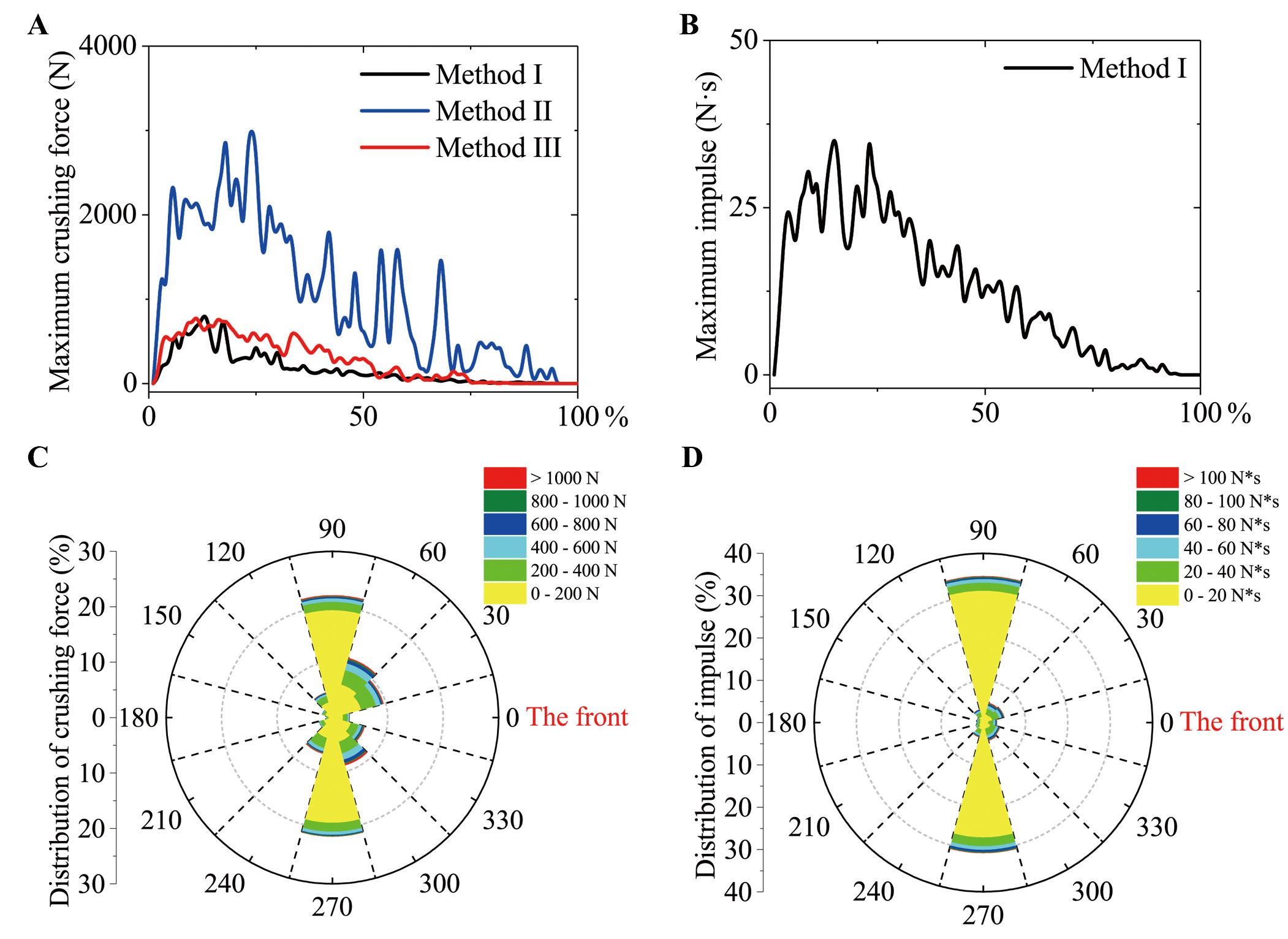


Fig. S3. Evacuation simulations in a single-exit room. The desired velocities of all individuals were 1.5 m/s. (A) The maximum contact force of all individuals throughout the entire evacuation process. Method I: The physiological acceleration limits of all individuals were assumed to be $a_{upper}=5 \text{m/s}^{2}$, and the physical contacts between individuals were modeled based on the collision impulses and pushing forces. Method II: The physiological acceleration limits of all individuals were assumed to be $a_{upper}=5 \text{m/s}^{2}$, and the physical contacts between individuals were modeled based only on the pushing forces. Method III: No physiological acceleration limits existed, and the physical contacts between individuals were modeled based only on the pushing forces. (B) The maximum collision impulse of all individuals throughout the entire evacuation process. (C) The distribution of the crushing force the individual was subjected to for all directions of the human body during an evacuation. (D) The distribution of the collision impulse the individual was subjected to in all directions of the human body during evacuation.

**
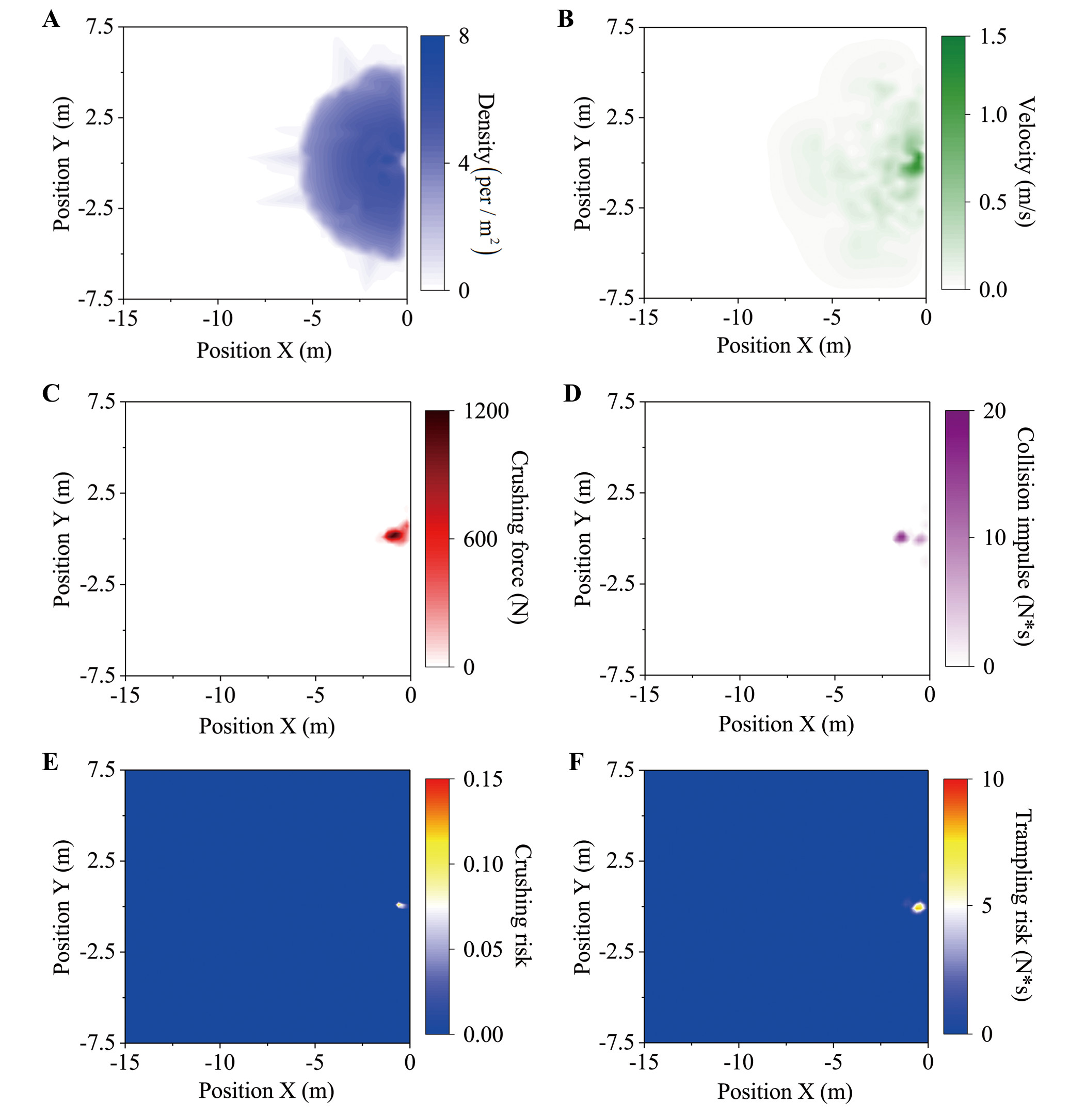
**

Fig. S4. The spatial distribution of the evacuation simulations in a single-exit room. The space was divided into grids of $0.2\times0.2 \text{m}$. (A) Local density of the crowd. (B) Instantaneous speed scalar value. (C) Scalar value of the crushing force the individual was subjected to. (D) Scalar value of the collision impulse the individual was subjected to. (E) The crushing risk for the individual. (F) The trampling risk of the individual.

**
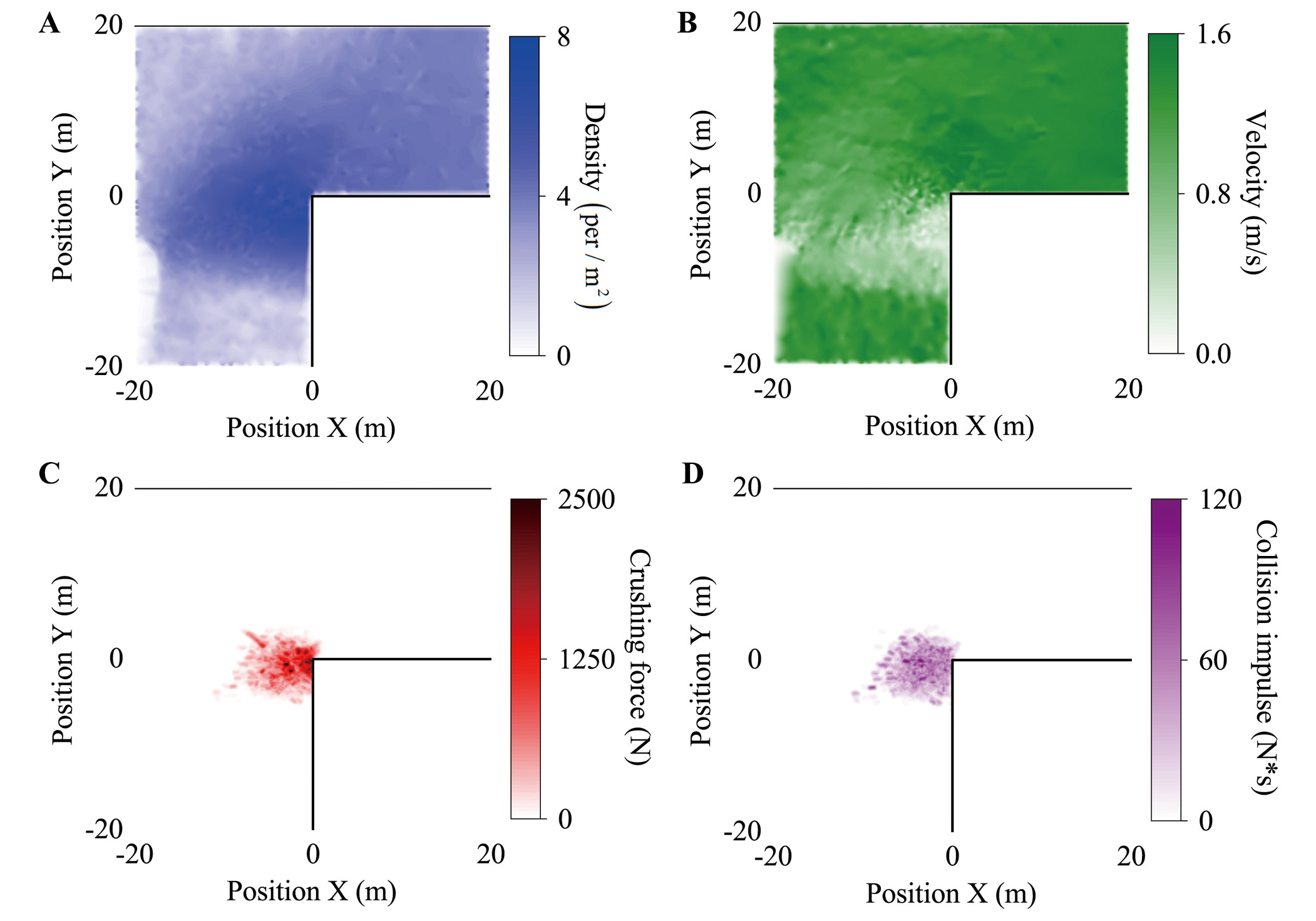
**

Fig. S5. The spatial distribution of the movement process of the Hajj pilgrimage crowd scene. The space was divided into the grids of $0.2\times0.2 \text{m}$. (A) Instantaneous local density of the crowd. (B) Instantaneous speed scalar value. (C) Scalar value of the crushing force the individual was subjected to. (D) Scalar of collision impulse the individual was subjected to.

**
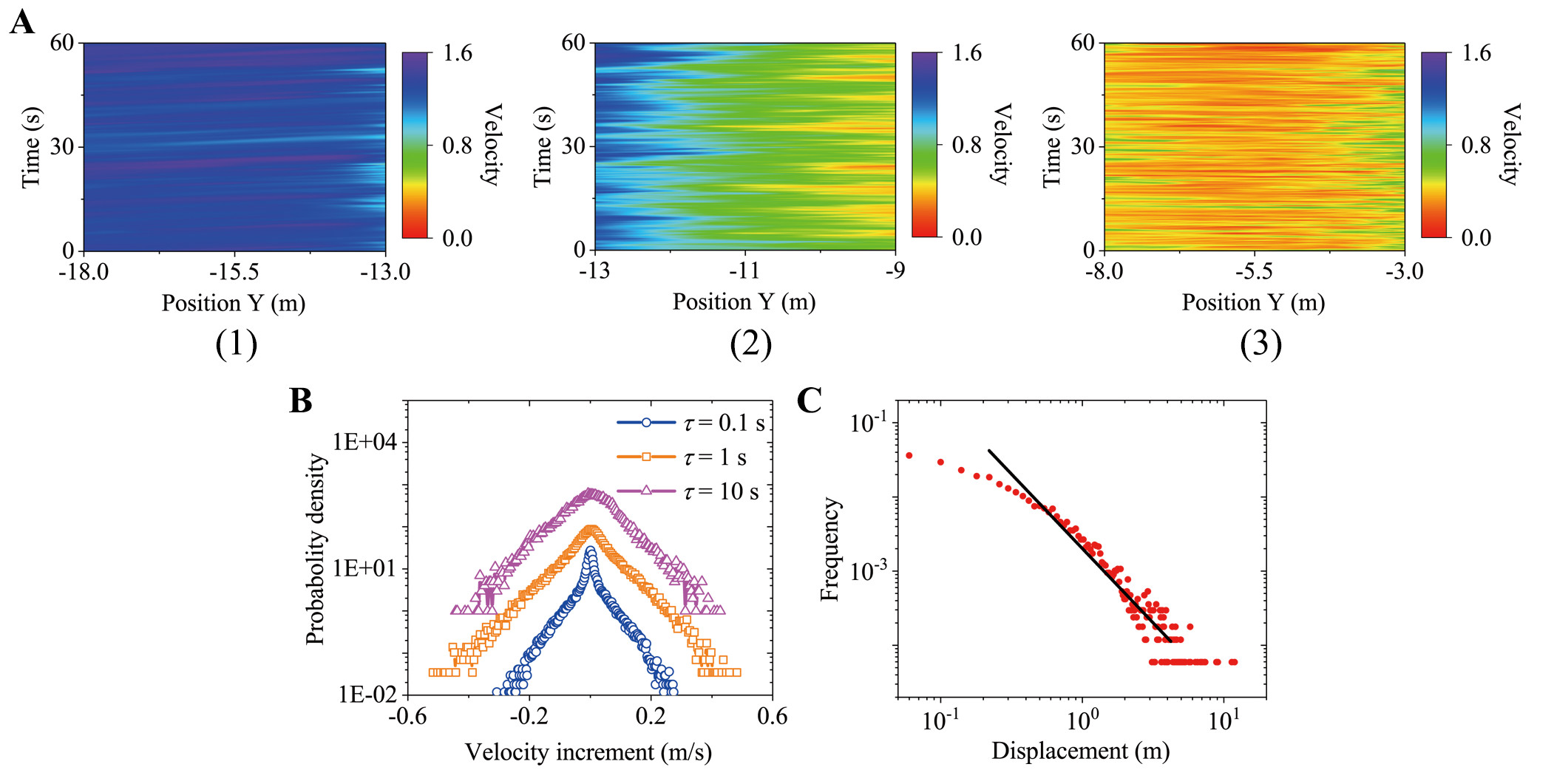
**

Fig. S6. The crowd motion state of the Hajj pilgrimage scene. (A) Typical space-time diagrams representing different kinds of collective motion. The color-coding indicates the local speed values (where pedestrians moved from the bottom up). (1) The diagram displays a smooth, laminar flow with occasional variations in speed. (2) The stop-and-go waves appear as they were empirically observed near the turn. (3) The average traffic flow was almost zero, but turbulent fluctuations in the flow occurred. (B) Probability density functions of the velocity increment in the turbulent regimes, determined over many locations, where *τ* =0.1 s (blue curve), *τ* =1 s (orange curve), and *τ* =10 s (pink curve). For clarity of presentation, the curves have been shifted in a vertical direction. The non-parabolic, peaked curve for small values of *τ*, which distinguishes turbulent from laminar flows, should be noted. (C) Distribution of displacements (location changes between subsequent stops, defined by $\left\|\vec{v}(\vec{r},t)\right\|<0.1 \text{m/s}$). The double-logarithmic representation reveals a power-law reminiscent of the Gutenberg-Richter law for earthquake amplitudes. This indicates that the individual could move only a very small distance at most moments, but the movement velocity was still not 0. Here, the slope is -2.00 ± 0.07, which is consistent with the empirical results of Helbing et al. (5)

**
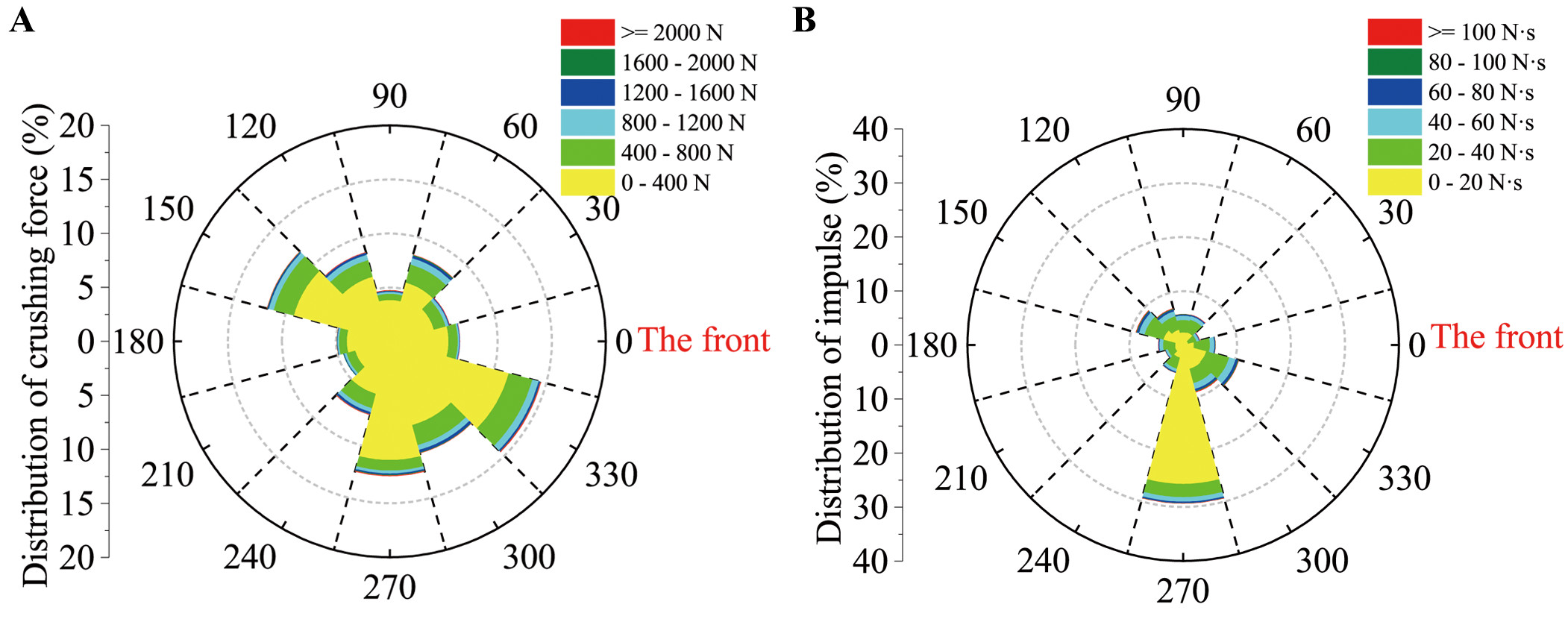
**

Fig. S7. Hajj pilgrimage scene within 15 s of the crowd movement process. (A) The distribution of the crushing force the individual was subjected to for all directions of the human body. (B) The distribution of the collision impulse the individual was subjected to for all directions of the human body.

**
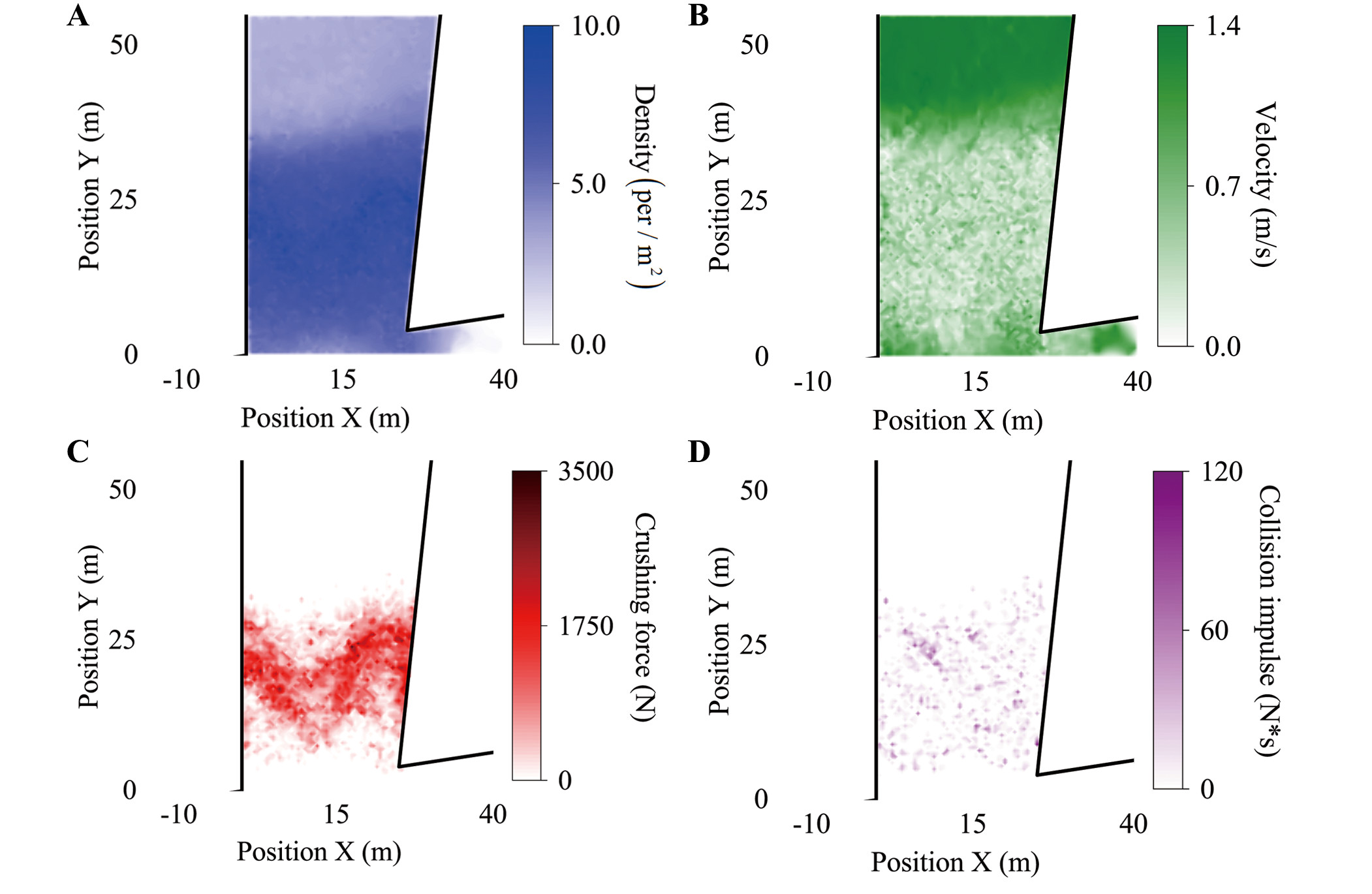
**

Fig. S8. The spatial distribution of the crowd motion process of the Love Parade simulation. The space was divided into the grids of $0.5\times0.5 \text{m}$. (A) Instantaneous local density of the crowd. (B) Instantaneous speed scalar value. (C) Scalar value of the crushing force the individual was subjected to. (D) Scalar value of the collision impulse the individual was subjected to.

**
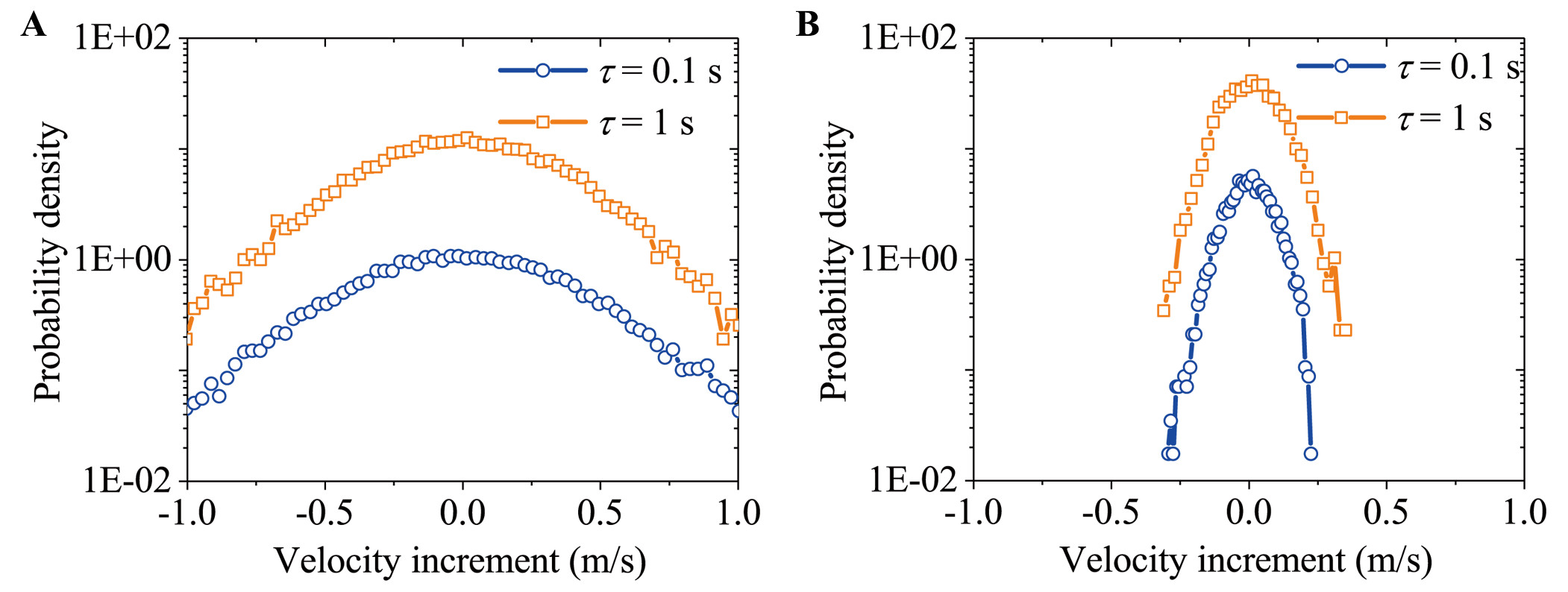
**

Fig. S9. Probability density functions of the velocity increment in the (A) laminar and (B) turbulent regimes, determined over many locations, with *τ* =0.1 s (blue curve) and *τ* =1 s (orange curve). For clarity of presentation, the curves have been shifted in a vertical direction. The non-parabolic, peaked curve for small values of *τ*, which distinguishes turbulent from laminar flows, should be noted.

**
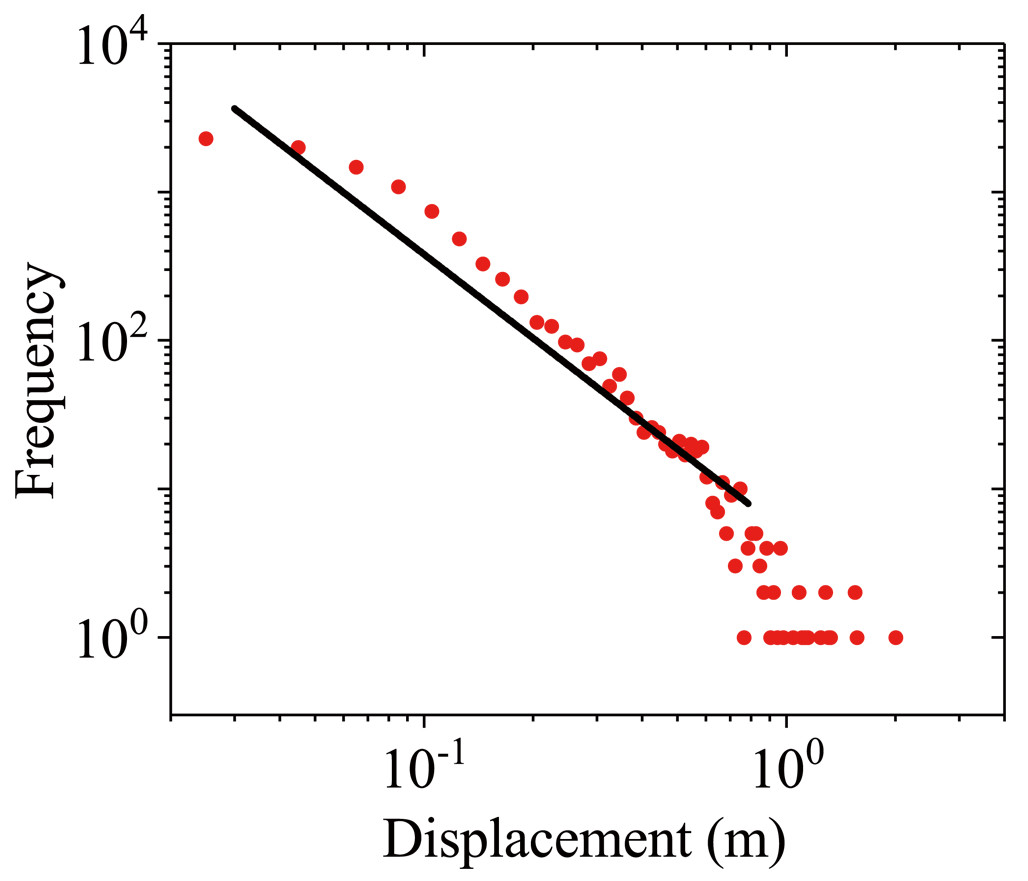
**

Fig. S10. Distribution of displacements (location changes between subsequent stops, defined by$\left\|\vec{v}(\vec{r},t)\right\|<0.04 \text{m/s}$). The double-logarithmic representation reveals a power law reminiscent of the Gutenberg-Richter law for earthquake amplitudes. Indicating that the crowd was in part of the local congestion, the individual could move only a very small distance at most moments, but the movement velocity was still not 0. Here, the slope is –1.88 ± 0.09, which is consistent with the empirical results of Ma et al. (6)

**
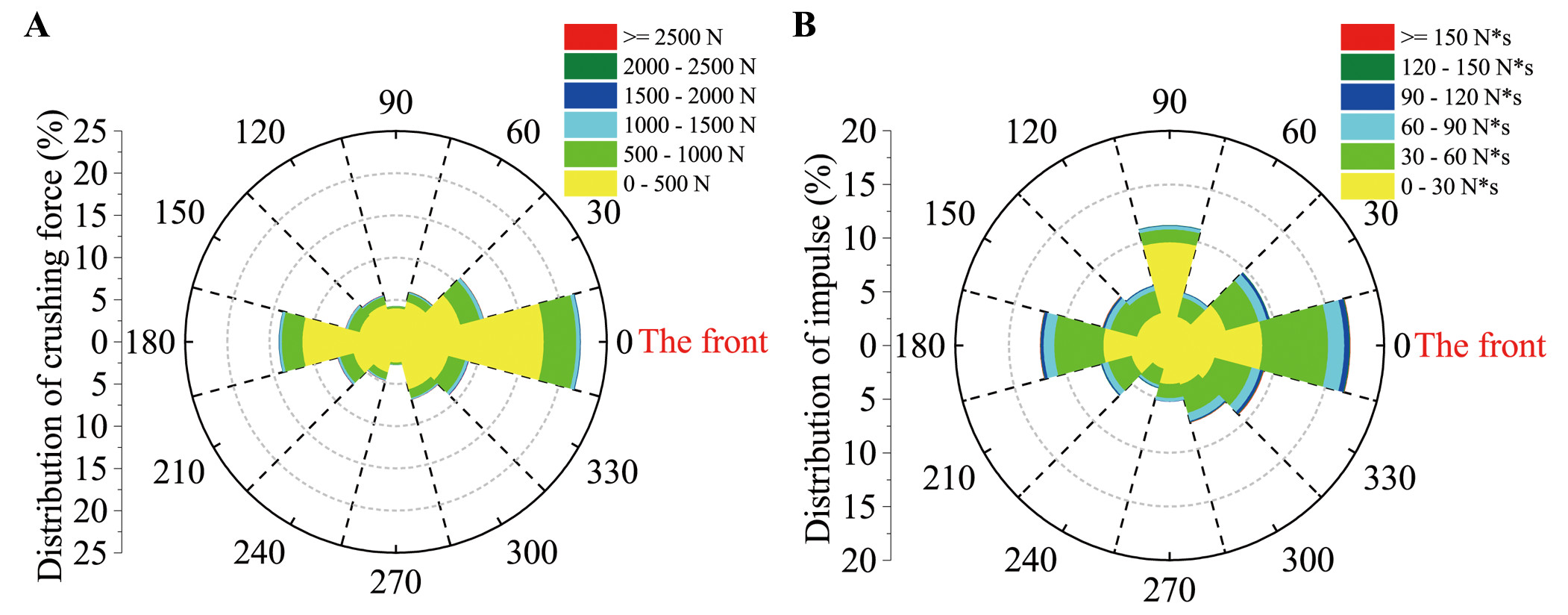
**

Fig. S11. Love Parade Simulation within 10 s of the crowd movement process. (A) The distribution of the crushing force the individual was subjected to for all directions of the human body. (B) The distribution of the collision impulse the individual was subjected to for all directions of the human body.

**References**

1. Van Leeuwen JMJ (2010) The domino effect. *American Journal of Physics* 78(7):721–727.

2. Kotelnikov E (2012) The Domino Effect. *Journal of Physical Science and Application* 2(6):195–199.

3. Kabalan B, Argoul P, Jebrane A, Cumunel G, Erlicher S (2016) A crowd movement model for pedestrian flow through bottlenecks. *Annals of Solid and Structural Mechanics* 8(1–2):1–15.

4. Lee RSC, Hughes RL (2006) Prediction of human crowd pressures. *Accident Analysis and Prevention* 38(4):712–722.

5. Helbing D, Johansson A, Al-Abideen HZ (2007) Dynamics of crowd disasters: An empirical study. *Physical Review E* 75(4):46109.

6. Ma J, Song WG, Lo SM, Fang ZM (2013) New insights into turbulent pedestrian movement pattern in crowd-quakes. *Journal of Statistical Mechanics: Theory and Experiment* 2013(2):P02028.
